# Thoracolumbar myelocele repair: how I do it

**DOI:** 10.1007/s00701-024-06163-2

**Published:** 2024-06-17

**Authors:** Nathalie Zimmermann, Mahmoud Messerer, Alberto Vandenbulcke

**Affiliations:** 1 Faculty of Biology and Medicine, University of Lausanne, Lausanne, Vaud, Switzerland; 2 Department of Neurosurgery, University Hospital of Lausanne, Lausanne, Vaud, Switzerland; 3 Unit of Pediatric Neurosurgery, University Hospital of Lausanne, Lausanne, Vaud, Switzerland

**Keywords:** Myelocele, Myelomeningocele, Open Spina Bifida, Spinal dysraphism, Congenital malformation surgical repair, Chiari II malformation, Hydrocephalus, Ventriculoperitoneal shunt

## Abstract

**Background:**

Myelocele is a rare form of open spina bifida. Surgical repair is recommended prenatally or in the first 48 h. In some cases, the repair may be delayed, and specific surgical factors need to be considered.

**Method:**

We give a brief overview of the surgical anatomy, followed by a description of the surgical repair of a thoracolumbar Myelocele in an 11-month-old child.

**Conclusion:**

Surgical repair of the Myelocele stabilizes the neurological status, prevents local and central nervous system infections. The understanding of Myelocele anatomy enables its removal while preserving as much healthy tissue as possible and restoring normal anatomy.

**Supplementary Information:**

The online version contains supplementary material available at 10.1007/s00701-024-06163-2.

## Key points

1. In the absence of life-threatening condition, any spinal dysraphism repair should be undertaken as soon as possible, ideally within the first 48 hours of life. However, circumstances may lead to a delayed repair, as in our case.

2. Each child should undergo a preoperative MRI of the spinal cord and imaging of the brain to look for signs of hydrocephalus (present in approximately 90% of cases).

3. A good anatomical understanding is fundamental.

4. The aim of the surgery is to restore the normal anatomical pattern, stabilize the neurological and clinical status of the child and prevent complications such as local infection or meningitis.

5. Microscopic magnification is crucial for identification of structures such as the opening of the normal dura into the skin, fusion of the spinal cord into the placode and the emergence of roots in epidural space.

6. Removal of the epithelium covering the placode is mandatory to prevent the formation of inclusion dermoid cysts.

7. Watertight closure of the dura mater is imperative to prevent CSF leakage.

8. Maximum effort should be made to facilitate primary closure of the soft tissue. Incision at the border of the dystrophic skin covering the MC and horizontal incision allows efficient skin transposition and closure.

9. Blunt dissection between the subcutaneous fat and the thoracolumbar fascia allows for the preservation of the perforating arteries.

10. Early signs of complications such as wound dehiscence, CSF leakage and infection of the operative site should be carefully monitored.

**Supplementary Information:**

The online version contains supplementary material available at 10.1007/s00701-024-06163-2.

## Relevant surgical anatomy

**Fig. 1:**
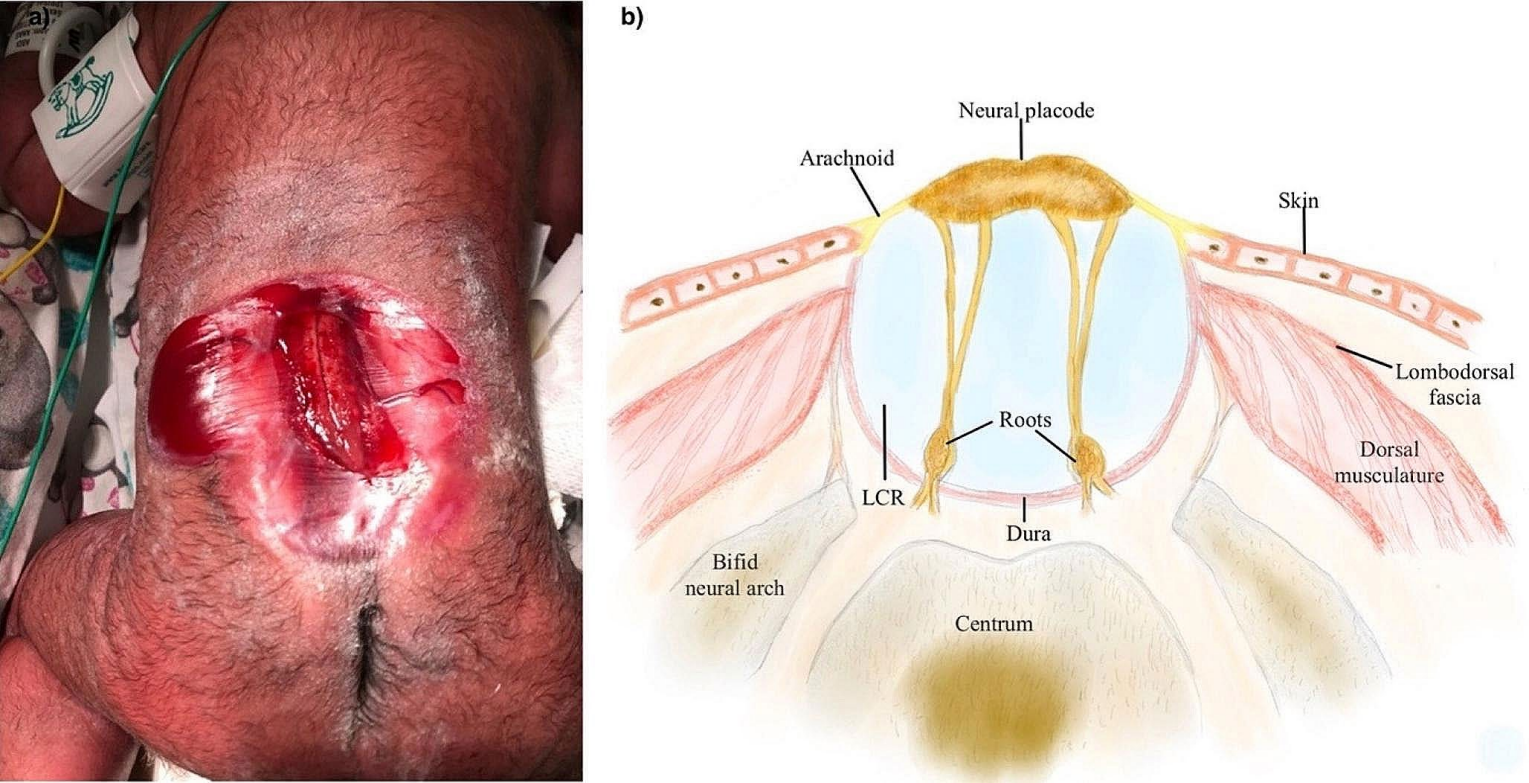
**(a)** Showing the Myelocele on the day of birth, the placode is visible at the center of the defect. **(b)** drawing representing a transverse view of a myelocele with anatomical surrounding relevant structures

A good understanding of normal embryological development is essential for comprehending anomalies in neural tube closure. Neural tube formation begins on the 17th day of gestation when the notochord triggers the ectoderm above into neuroectoderm, initiating the formation of the neural groove along the embryo’s dorsal midline. Neural folds on both sides of the groove rise up and converge, merging their cells dorsally to create a hollow tube. Closure of the neural tube begins at the cervical region and closes in opposite directions [1]. The malformed spinal cord or primitive neural plaque (placode) appears as a flattened neural tissue, with its borders extending laterally with the malformed meningeal layers (Fig. 1).

The outer or dorsal surface of the placode represents what would be the spinal ependymal layer, while the inner or ventral surface corresponds to the external pial surface of the spinal cord. In the case of myelocele (MC), the placode is exposed through the spinal dysraphism without the posterior expansion of the subarachnoid space typical of the myelomeningocele.

**Fig. 2:**
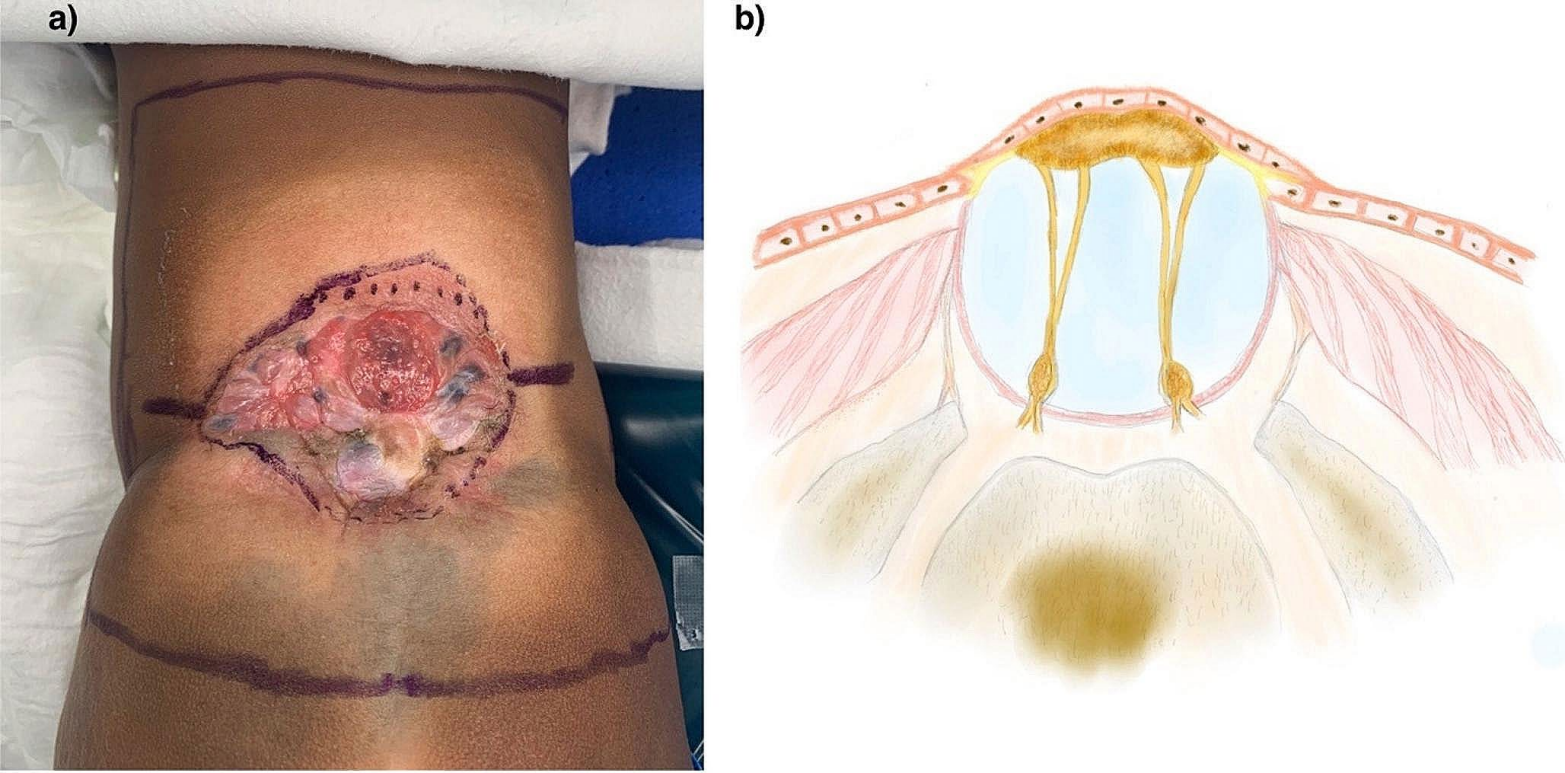
**(a)** Showing the photo on operation day, the placode and the surrounding defect have been covered by a layer of dystrophic skin. The incision is marked all around the dystrophic skin with two lateral incisions to increase skin mobility. **(b)** A drawing representing a transverse view of the myelocele covered by the dystrophic layer of skin

The residual neural function of the placode is highly debated [2]. If the neural defect is not immediately repaired, partial epithelialization of the placode makes the placode identification and dissection of its boundaries with the skin more complex (Fig. 2). As a result of the disrupted neurulation process, both ventral and dorsal spinal roots come out from the ventral side of the placode. The dorsal roots exit laterally compared to the ventral ones. Thus, the dorsal roots originate in the area where the placode continues in the arachnoid membrane, known as the junctional zone (Fig. 1) [6]. The dura, which contains the placode, is usually enlarged and fused with the skin on the back. Dysgenesis of the two lateral growth centers of the vertebral bodies, causes an outward rotation of the pedicles and results in a spinal canal that is wider and shallower than normal, lacking dorsal bony elements [5]. The quality of the skin coverings varies depending on the re-epithelization process related to surgical repair delay [4].

## Description of the technique (video)

The aim of the surgical repair is to recreate the normal embryological pattern of development. To achieve this, you should isolate the unmerged segment, reconstruct the original neural tube shape, and then close over with the dura mater and soft tissues. Under general anesthesia, the patient is settled in the prone position. A special anti-skid polyurethane foam pad is used to prevent bedsores or any intraoperative nerve injuries. Rolls and additional gel pads are placed under the chest and hips to prevent abdominal compression.

Intravenous Cefazoline, at a dosage of 30 mg/kg, is administered preoperatively and the dose is repeated after four hours. The thoracolumbar area is cleaned with Povidone iodine scrub, taking care to avoid rubbing over the placode. The skin is disinfected using a 10% Povidone-iodine aqueous solution, and a 5% solution is used for the skin covering the placode.

**Fig. 3:**
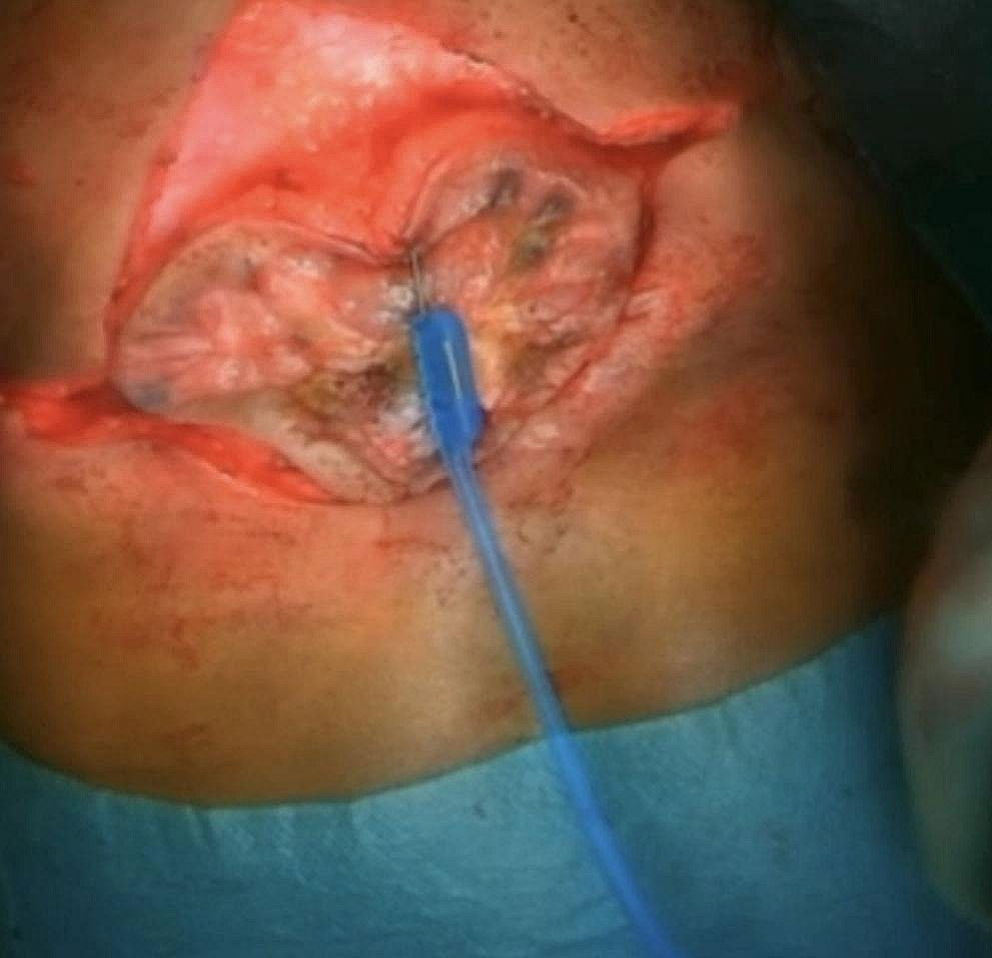
Intraoperative view after skin incision all around the borders of the posterior defect and identification of the thoracolumbar fascia

After disinfecting and draping the skin, an incision is made all around the boundary between the dystrophic skin covering the affected area and the healthy skin. Two additional horizontal incisions are made on both sides to allow for better mobility of the skin during closure (Fig. 3).

**Fig. 4:**
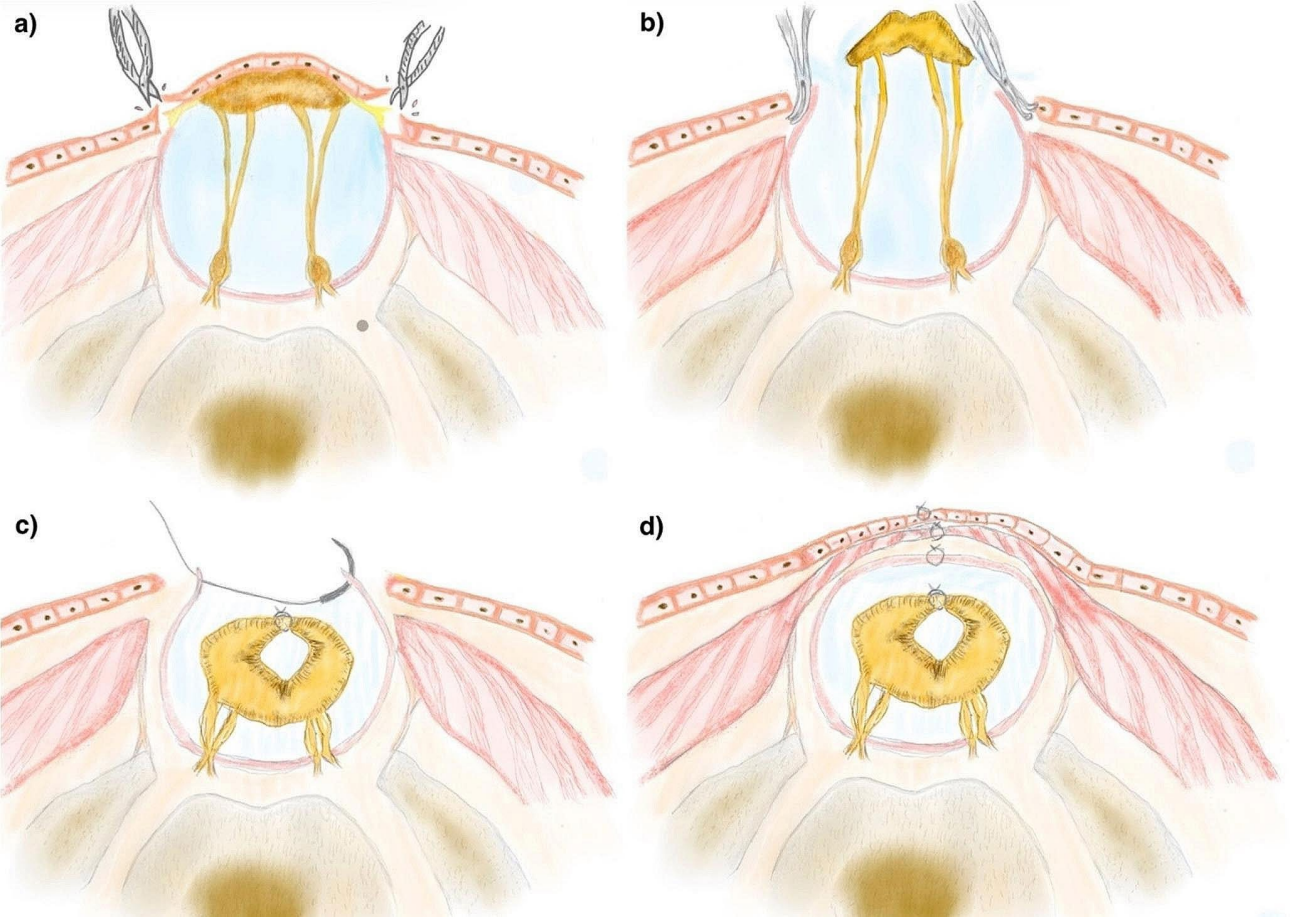
Drawing showing **(a)** the progressive dissection of the placode from normal skin and **(b)** from the superficial dystrophic skin and the surrounding tissues. **c-d)** Layer-by-layer closure to restore the most normal anatomy possible and ensure the reconstruction of the neural tube, watertight dural closure and coverage with the superficial soft tissues

**Fig. 5:**
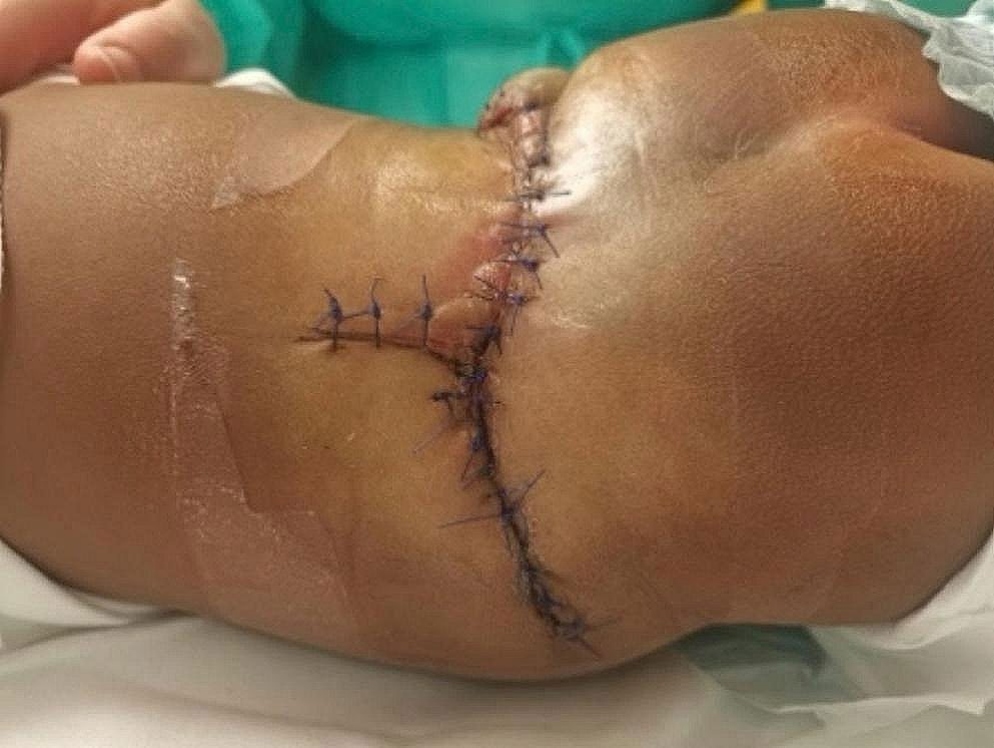
Figure showing wound closure at postoperative day 2, with no tension on the skin flaps

The thoracolumbar fascia is identified all over the defect. The fascia is incised cranially on the midline over the spinous process of the last normal vertebra, and the muscles are dissected subperiosteally from the laminae. Under microscopic magnification, we identify the normal dura mater under the most caudal lamina which is typically not fully fused. The dura is dissected until its opening to the skin where the placode is enclosed. Microsurgical dissection and excision of the epithelial tissue covering the placode is performed using an ophthalmic scalpel once the point where the dura merges with the skin is identified all around the defect (Fig. 4a and b). Epidural space dissection is performed to free the dura from paravertebral muscles and fascia, and open dysplastic lamina remnants to ensure a relaxed and watertight covering of the placode. The placode is now fully exposed within the dura mater, and careful dissection of the arachnoidal adhesions between the placode and dura mater is performed, making sure to pay attention to the spinal root on the ventral side of the placode. Placode reconstruction in a tube shape is completed with separated non-absorbable stitches. Finally, the dura mater is securely closed with separated non-absorbable stitches to ensure a watertight closure (Fig. 4c). Blunt dissection between the subcutaneous fat and the thoracolumbar fascia allows for skin mobilization and helps avoid excessive tension during skin closure. A vertical midline incision is performed to increase skin rotation. Subcutaneous closure is performed with anchor points to the fascia to reduce surface tension. Single nonabsorbable stitches are used for skin closure (Figs. 4d and 5).

### Indications

The aim of the surgery for MC is to stabilize the neurological and clinical status of the child and prevent complications such as local infection or meningitis. Typically, the surgery is performed in utero or within the first 48 hours of life. Any delay in repair increases the risk of infections and exposes to a higher risk of neurological function deterioration [2]. In cases of extreme deferred repair, such as our case, prevention of neurological functions is obsolete and emphasis is placed on infection prevention.

### Limitations

Life-threatening co-existing malformation is the only current ethical-legal contraindication to provide surgical treatment. Some severe forms with multi-segment, large lesions associated with vertebral anomalies can be technically challenging [1]. Extremely large superficial tissue defects can be a limitation for optimal surgical repair, possibly requiring repair in several stages. In case of central nervous system or local infection, surgical repair should be postponed until the infection has been resolved [2, 5].

## How to avoid complications

When performing a delayed repair, it is essential to meticulously remove the squamous epithelial layer over the placode to prevent the formation of inclusion dermoid cysts [8]. Due to the low chance of residual neurological function preservation, epithelial removal should be privileged over placode preservation. However, efforts should still be made to preserve at least a portion of the placode. Particular emphasis should be placed on tubulation of the placode to reduce risks of adherence of the spinal cord to the spinal canal and secondary tethered cord. However, dissection of the ventral placode is at risk of spinal root injury and should be performed under microscopic magnification. It is crucial to ensure a watertight closure of the dura mater to prevent cerebrospinal fluid (CSF) leakage. Thus, epidural dissection is fundamental to adequately mobilize and close the dura. It is important to prepare and approximate the skin to avoid tension during closure, as this is crucial for good wound healing. The incision should be made on the healthy skin at the border with the soft tissue defect (Figs. 2 and 3). The horizontal incisions allow for effective skin transposition for primary closure. Moreover, to preserve good vascular perfusion, dissection between the subcutaneous fat and thoracolumbar fascia should be performed with specific attention to preserve perforating arteries. Due to the frequent association with hydrocephalus, ventriculoperitoneal shunt should be evaluated pre-operatively to reduce the risk of CSF leak [2, 5].

In cases where the patient retains neurological functions such as leg movement or sphincter function, it is crucial to use intraoperative neuromonitoring [3]. In our cases, no evidence of residual function was observed pre-operatively and no neuromonitoring was performed.

## Specific perioperative considerations

### Pre-operative workup

A good clinical neurological examination is essential to document the level of neurological damage. The urodynamic test is recommended to evaluate residual bladder function. Prior to surgery, every child should undergo a brain and spine MRI to check for hydrocephalus (present in approximately 90% of cases) [1, 7], associated anomalies such as Chiari II malformation, and improve anatomical comprehension of the neural defect. Any central nervous system or local infection should be ruled out before the procedure.

### Postoperative workup

In the postoperative period, it is recommended to closely monitor patients in intensive or intermediate care units for possible signs of intracranial hypertension or CSF leak. It is preferable a lateral or prone position to discharge any tension over the wound, ideally for the first 5 postoperative days [5]. No drain is placed into the wound to avoid a CSF leak. Close monitoring of wound healing is recommended during the first week. Immediate post-operatively imaging is usually unnecessary. A spinal MRI can be performed a few months after surgery to assess the reconstruction and monitor the risk of tethering and dermoid cysts.

## Specific information to give to the patient about surgery and potential risks

The most common complication is wound breakdown, which is often caused by CSF leakage, excessive tension, or skin flap devascularization. These factors are known to have a negative impact on wound healing [2]. For large skin defects, the risk of wound healing failure is significant. Defects larger than 20-25cm^2^ may require plastic reconstruction surgery [2, 5].

Conversely, wound infection is a far less common complication following MC repair, occurring in less than 2% of procedures [6]. For children with remaining preoperative neurological functions, parents should be informed about the potential risk of spinal nerve roots injury during the procedure, which may lead to new impairment.

## Electronic supplementary material

Below is the link to the electronic supplementary material.

Supplementary Material 1

## Data Availability

Not applicable.
